# Different effects of methylphenidate and atomoxetine on the behavior and brain transcriptome of zebrafish

**DOI:** 10.1186/s13041-020-00614-4

**Published:** 2020-05-06

**Authors:** Shiho Suzuki, Ryo Kimura, Shingo Maegawa, Masatoshi Nakata, Masatoshi Hagiwara

**Affiliations:** 1 Department of Anatomy and Developmental Biology, Graduate School of Medicine, Kyoto University, Kyoto, 606-8501 Japan; 2 Department of Intelligence Science and Technology, Graduate School of Informatics, Kyoto University, Kyoto, 606-8501 Japan

**Keywords:** Attention deficit-hyperactivity disorder (ADHD), Behavior, Lipid metabolism, Methylphenidate, Atomoxetine, Zebrafish, Transcriptome

## Abstract

Attention deficit-hyperactivity disorder (ADHD) is a prevalent neuropsychiatric disorder found in children. It is characterized by inattention, hyperactivity, and impulsivity. Methylphenidate (MPH) and atomoxetine (ATX) are commonly prescribed for the treatment of ADHD. In the present study, we examined the behavioral and brain transcriptome changes in MPH-treated and ATX-treated zebrafish. In behavioral analysis, zebrafish showed opposite response to each treatment. MPH-treated fish showed higher anxiety-like behavior while ATX-treated fish showed lower anxiety-like behavior. Further, we performed RNA sequencing analysis of zebrafish brain to elucidate the underlying biological pathways associated with MPH and ATX treatment. Interestingly, we found that shared differentially expressed genes in MPH-treated and ATX-treated fish were instrumental in cholesterol biosynthesis pathway and were regulated in opposite manner. Our findings highlight the contrast between MTH and ATX, and may suggest the alterations in clinical practice for these medications and drug development for ADHD.

Attention deficit-hyperactivity disorder (ADHD), which is a common childhood neuropsychiatric disorder, affects around 5% in children globally. It is characterized the by symptoms of inattention and/or hyperactivity-impulsivity [1]. Currently, pharmacological intervention is considered effective for patients with ADHD; in particular, methylphenidate (MPH) and atomoxetine (ATX) are the main medications being prescribed for ADHD treatment [2]. Previous studies have suggested that MPH inhibits the reuptake of norepinephrine and dopamine into presynaptic neurons [2], whereas, ATX is thought to inhibit presynaptic norepinephrine transporter selectively with secondary effects on dopaminergic systems [2]. However, the underlying mechanisms for the amelioration of clinical symptoms in ADHD patients using these medications are obscure. Moreover, these medications do not exert similar effect in all patients with ADHD [3]. Further, there is a growing concern about the adverse effects of MPH with prolonged use, including an increased risk of psychosis [4]. Therefore, it is necessary to elucidate the effects of these medications on brain and behavior using animal models.

Zebrafish are popular experimental animals, which have recently attracted attention in the field of neuroscience [5]. In addition to their cost-effectiveness, zebrafish have > 80% genes in common with humans and other animals with similar brain organization. Further, due to the simplicity of assessing behavioral changes, zebrafish have the potential to unravel the complexity of the neuropsychiatric disorders such as epilepsy, and help in the development of new therapies [6, 7]. To the best of our knowledge, there is no published research known to have examined the effect of ADHD medications on both brain and behavior under the same experimental conditions.

In the present study, we aimed to assess the effects of MPH and ATX on brain through gene expression profiles and behavioral analysis using zebrafish. To decide the dose of MPH and ATX, initially, the fish were exposed to different doses of each drug for 4 h (Additional file 5: Figure S1). The minimum dose that altered the behavior was adopted for further analysis using AB wild-type strain fish (10 mg/L for MPH, and 3 mg/L for ATX). Further, behavior and transcriptome analyses were conducted on these fish. Briefly, 4-h exposure of MPH and ATX caused changes in behavior, which were similar to 8-day exposure (description follows); however, the results of transcriptome analyses were less significant (Additional file 6: Figure S2, Additional file 3: Table S3, and Additional file 4: Table S4). Therefore, in order to determine the effect of exposure for longer term, we conducted the novel tank test and transcriptome analyses on MPH- or ATX- treated zebrafish for 8 days. In this report, we have presented and discussed the results of the analyses following 8-day exposure.

To investigate the behavioral effects due to these medications, zebrafish were exposed to MPH (10 mg/kg) or ATX (3 mg/kg) for 8 days, followed by behavioral analysis using the novel tank test (Fig. 1a). Representative traces of the fish in each group (control, MPH-treated, and ATX-treated fish) are shown in Fig. 1b-d. MPH-treated fish spent significantly less time in top area and more time in bottom area (Fig. 1e, f). In contrast, ATX-treated fish spent significantly more time in top area and less time in bottom area (Fig. 1e, f). These contrasting findings may provide insight into the characteristics and effects of each medication on the brain.

**Fig. 1:**
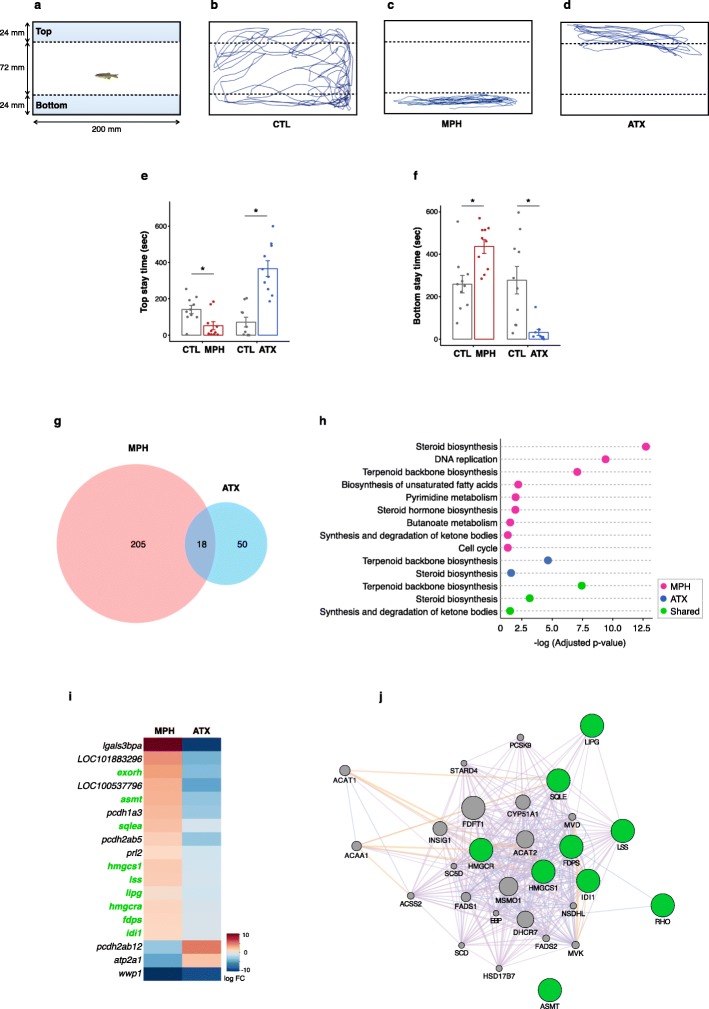
(**a-f**): Behavioral effects of methylphenidate (MPH) and atomoxetine (ATX) treatment for 8 days in zebrafish. Novel tank test was performed in 10 fish per group for 10-min period. **a**: Schematic representation of novel tank test. **b-d**: Representative traces of zebrafish in Control (CTL) **(b)**, MPH-treated **(c)**, and ATX-treated fish **(d)**. **e**: Time spent in top area (s). **f**: Time spent in bottom area (s). Student’s t-test was performed for statistical analysis and asterisk denotes *p* < 0.05. **(g-j)**: Differentially expressed genes (DEGs) in the brains of zebrafish treated with MPH or ATX. **g**: Venn diagram shows the total number of DEGs among controls, MPH-treated and ATX-treated fish. Analysis of differential expression in each cohort resulted in 223 DEGs between MPH-treated and controls, and 68 DEGs between MPH-treated and controls. Comparison of the MPH- and ATX-treated fish to the controls resulted in 18 shared genes with significant differential expression. The DEGs were defined as genes with false discovery rate of less than 0.25 and absolute value of fold change larger than 1.2. **h**: KEGG pathway analysis for each DEG. Significantly enriched pathways are shown for DEGs in each group (adjusted *p*-value< 0.05). DEGs in MPH-treated fish are shown in pink, ATX-treated fish in blue, and shared DEGs between MPH- and ATX-treated fish in green. **i**: Heatmap shows shared DEGs between MPH- and ATX-treated fish (rows) and drug exposure (columns). Genes that have human orthologs are highlighted in green. Blue represents down-regulated expression, red represents up-regulated expression, and white represents no change in expression. **j**: Network analysis for shared DEGs which have human orthologs. The zebrafish gene names were converted to the human names. Green nodes represent the query and grey nodes represent the results. Network was drawn based on co-expression (purple edges), predicted networks (orange edges), and co-localization (blue edges)

Further, we performed RNA sequencing (RNA-Seq) analysis to evaluate the transcript profile of the brain. RNA samples with RNA integrity number ≥ 8 were used; and quality did not differ significantly among the groups. We identified 223 genes whose expression was significantly different between MPH-treated and control fish; and 68 genes that showed significantly different expression levels between ATX-treated and control fish (Fig. 1g and Additional file 1: Table S1). Comparison of the differentially expressed genes (DEGs) among the groups revealed that 18 genes were significantly different between controls and both MPH-treated and ATX-treated fish (Fig. 1g). Further, we performed Kyoto encyclopedia of genes and genomes (KEGG) pathway analysis to elucidate the underlying biological pathways comprising the DEGs [8]. We found that the DEGs were significantly involved in steroid biosynthesis and terpenoid backbone biosynthesis in both groups, suggesting that MPH and ATX treatment might affect pathways involved in lipid metabolism (Fig. 1h and Additional file 2: Table S2).

Further, we assessed whether the shared DEGs in MPH- and ATX-treated fish were up-regulated or down-regulated. We found that 17 out of 18 shared DEGs showed opposite trend in MPH-treated and ATX-treated fish (Fig. 1i). Among 18 DEGs, 9 (*exorh*, *asmt*, *sqlea*, *hmgcs1*, *lss*, *lipg*, *hmgcra*, *fdps*, and *idi1*) had an ortholog in human. Finally, we identified the interaction network, generated by GeneMANIA, which closely connected 8 out of 9 human ortholog genes (Fig. 1j). Notably, *FDPS*, *HMGCR*, *HMGCS1*, *IDL1*, *LIPG*, *LSS*, and *SQLE* genes are known to be major factors in human cholesterol biosynthesis pathway.

In the present study, we examined the effect of MPH and ATX on brain transcriptome and behavior in zebrafish, and identified several DEGs and the associated pathways. The novel tank test, which is based on the natural instinct to seek protection in an unfamiliar environment, is a popular assay to assess the anxiety-like behavior in zebrafish [5]. Usually, an increase in exploration occurs as the fish gradually acclimates to the new environment [5]. We found that MPH-treated fish spent more time in bottom area, while ATX-treated fish spent more time in top area. These findings suggest that MPH exposure causes higher anxiety-like levels, while ATX exposure causes lower anxiety-like levels. Previous studies have also reported that rats exposed to MPH exhibited anxiety-like behavior [9, 10]; however, no animal study has yet examined the relationship between ATX exposure and anxiety-like behavior.

Transcriptome analysis is a fruitful approach to gain a comprehensive insight into the biological pathways potentially affected by diseases or medications. In this study, RNA-seq analysis of zebrafish brain was used to identify the pathways associated with the ADHD medications. Interestingly, most of the shared DEGs in MPH-treated and ATX-treated fish were regulated in opposite directions, which supports the data of our behavioral analysis. Pathway analysis of the DEGs showed that they were involved in lipid metabolism. Previous studies involving human patients and polychlorinated biphenyls-induced rats have shown significant association between dysregulated genes and lipid metabolism, including triglyceride lipase activity and peroxisome proliferator-activated receptor α (PPARα) [11–13]. Altered lipid metabolism has been reported in a range of neurological and psychiatric disorders [14]. In addition, children with ADHD have been reported to have lower levels of n-3 polyunsaturated fatty acids than healthy controls, although it remains unclear whether it is due to poor nutritional intake or altered lipid metabolism [14, 15].

To the best of our knowledge, this is the first study to compare MPH and ATX in terms of their effect on brain transcriptome and behavior. However, our study has a few limitations. First, the direct causal relationship between altered behavior and lipid metabolism in brain has not been identified. Second, dopamine and norepinephrine-related genes, which may play an important role in pharmacokinetics of ADHD drugs, were not observed in DEGs identified in this study. Third, the effect of time on MPH and ATX treatment needs to be further assessed. Although 4-h treatment resulted in behavioral changes similar to those of 8-day treated fish, 4-h exposure did not result in significant changes in the transcriptome. Future research on the effects of the drugs for different time durations may help to address this point. Although further investigations are required, our findings shed light on the necessity to rethink the strategies for drug development and clinical application of ADHD medications.

## Supplementary information

**Additional file 1: Table S1.** Differential expression profiles for 8-day exposure.

**Additional file 2: Table S2.** KEGG pathway analysis for 8-day exposure.

**Additional file 3: Table S3.** Differential expression profiles for 4-h exposure.

**Additional file 4: Table S4.** KEGG pathway analysis for 4-h exposure.

**Additional file 5: Fig. S1.** Dose-response curve of methylphenidate and atomoxetine.

**Additional file 6: Fig. S2.** The effect on behavior and transcriptome after 4 h of treatment of methylphenidate and atomoxetine.

**Additional file 7:** Materials and Methods

## Data Availability

The datasets and computer code used in the current study are available from the corresponding author on reasonable request. Methods are presented in Additional file 7.

## Abbreviations

ADHD: Attention deficit-hyperactivity disorder
ATX: Atomoxetine
CTL: Control
DEGs: Differentially expressed genes
KEGG: Kyoto encyclopedia of genes and genomes
MPH: Methylphenidate
RNA-Seq: RNA sequencing
