# Supplementary material for: Different effects of methylphenidate and atomoxetine on the behavior and brain transcriptome of zebrafish
Source: Mol Brain. 2020 May 6;13:70. doi: 10.1186/s13041-020-00614-4 (PMC7203832; doi:10.1186/s13041-020-00614-4)
Supplement: Supplementary file 1 — Additional file 1: Table S1. Differential expression profiles for 8-day exposure. [file 13041_2020_614_MOESM1_ESM.docx]

**Table S1.** **Differential expression profiles for 8-day exposure.**

| **MPH for 8 days** |  |  |  |  | **ATX for 8 days** |  |  |  |
| --- | --- | --- | --- | --- | --- | --- | --- | --- |
| **Gene Symbol** | **logFC** | **P-value** | **FDR** |  | **Gene Symbol** | **logFC** | **P-value** | **FDR** |
| seph | -8.78 | 6.57E-17 | 2.50E-12 |  | pcdh2ab12 | 4.93 | 3.98E-55 | 1.51E-50 |
| wwp1 | -10.46 | 8.01E-12 | 1.52E-07 |  | lgals3bpa | -9.92 | 1.43E-23 | 2.72E-19 |
| si:dkey-26g8.4 | -9.97 | 1.32E-11 | 1.68E-07 |  | pcdh10a | -1.28 | 1.05E-20 | 1.34E-16 |
| sqlea | 2.34 | 5.71E-11 | 5.43E-07 |  | LOC101883687 | 9.46 | 4.45E-15 | 4.24E-11 |
| pcdh2ab9 | -1.98 | 2.78E-10 | 2.11E-06 |  | si:ch211-194e18.2 | 1.26 | 9.86E-14 | 7.50E-10 |
| msmo1 | 1.34 | 1.91E-09 | 1.21E-05 |  | LOC100004091 | 3.61 | 2.63E-12 | 1.67E-08 |
| hmgcs1 | 1.85 | 5.48E-09 | 2.98E-05 |  | pbx3b | 0.80 | 2.93E-09 | 1.59E-05 |
| myhz1.1 | -11.77 | 6.34E-09 | 3.01E-05 |  | LOC101884148 | 8.36 | 4.83E-09 | 2.30E-05 |
| pcdh2ab5 | 1.67 | 1.08E-08 | 4.57E-05 |  | LOC100006428 | -8.55 | 7.60E-09 | 3.21E-05 |
| cyb5r2 | 2.35 | 1.27E-08 | 4.85E-05 |  | slc25a18 | -1.19 | 2.50E-08 | 9.50E-05 |
| araf | -1.06 | 2.77E-08 | 8.82E-05 |  | ociad2 | -9.38 | 8.66E-08 | 2.99E-04 |
| cyp51 | 1.44 | 2.78E-08 | 8.82E-05 |  | rcvrna | -8.16 | 9.43E-08 | 2.99E-04 |
| lyz | 2.60 | 4.68E-08 | 1.37E-04 |  | si:ch211-244b2.1 | 0.83 | 4.06E-07 | 1.19E-03 |
| LOC103912038 | -8.46 | 5.23E-08 | 1.42E-04 |  | LOC103911039 | -8.83 | 7.03E-07 | 1.91E-03 |
| nsdhl | 1.93 | 8.01E-08 | 1.99E-04 |  | ntd5 | 1.03 | 1.51E-06 | 3.84E-03 |
| bhmt | 0.77 | 8.37E-08 | 1.99E-04 |  | urp2 | 1.22 | 1.81E-06 | 4.31E-03 |
| si:ch73-236c18.7 | 4.53 | 1.24E-07 | 2.77E-04 |  | zgc:92630 | -0.88 | 2.24E-06 | 5.01E-03 |
| lgals3bpa | 10.49 | 1.40E-07 | 2.97E-04 |  | rdh5 | -8.47 | 4.30E-06 | 8.99E-03 |
| irbp | 3.22 | 1.89E-07 | 3.78E-04 |  | crx | -7.51 | 4.49E-06 | 8.99E-03 |
| lss | 1.77 | 2.19E-07 | 4.18E-04 |  | exorh | -3.87 | 4.91E-06 | 9.35E-03 |
| hmgcra | 1.46 | 2.55E-07 | 4.61E-04 |  | LOC101885578 | 8.61 | 5.80E-06 | 1.05E-02 |
| LOC101884724 | 10.62 | 2.83E-07 | 4.90E-04 |  | LOC101885914 | -7.66 | 6.29E-06 | 1.09E-02 |
| fdps | 1.33 | 3.09E-07 | 5.04E-04 |  | si:dkey-21o22.2 | -7.77 | 1.16E-05 | 1.91E-02 |
| pcdh2ab12 | -3.31 | 3.23E-07 | 5.04E-04 |  | proca1 | -1.47 | 1.34E-05 | 2.09E-02 |
| sc5d | 1.22 | 3.31E-07 | 5.04E-04 |  | gngt2a | -7.21 | 1.37E-05 | 2.09E-02 |
| wwp2 | -0.72 | 3.44E-07 | 5.04E-04 |  | prl2 | -1.19 | 1.50E-05 | 2.20E-02 |
| idi1 | 1.29 | 6.92E-07 | 9.71E-04 |  | LOC402866 | -1.43 | 1.63E-05 | 2.29E-02 |
| qkia | -1.64 | 7.15E-07 | 9.71E-04 |  | slc16a6b | -1.28 | 1.69E-05 | 2.30E-02 |
| LOC103909038 | -8.07 | 7.93E-07 | 1.04E-03 |  | ntn1b | 0.70 | 1.77E-05 | 2.33E-02 |
| rcan3 | -1.32 | 1.39E-06 | 1.76E-03 |  | zgc:55943 | -1.04 | 2.27E-05 | 2.84E-02 |
| mibp | 0.92 | 1.73E-06 | 2.12E-03 |  | myo15aa | -7.50 | 2.31E-05 | 2.84E-02 |
| fdft1 | 1.44 | 1.98E-06 | 2.35E-03 |  | hmgcs1 | -1.14 | 3.67E-05 | 4.36E-02 |
| exorh | 3.64 | 2.58E-06 | 2.98E-03 |  | zgc:55888 | -7.65 | 4.05E-05 | 4.67E-02 |
| kcnt2 | 1.47 | 3.01E-06 | 3.36E-03 |  | lss | -0.93 | 5.00E-05 | 5.49E-02 |
| rrm2 | 0.91 | 3.19E-06 | 3.46E-03 |  | lipg | -1.20 | 5.05E-05 | 5.49E-02 |
| aacs | 0.96 | 3.32E-06 | 3.51E-03 |  | bsk146 | -0.95 | 7.63E-05 | 7.94E-02 |
| histh1l | -1.43 | 3.52E-06 | 3.57E-03 |  | asmt | -3.45 | 7.72E-05 | 7.94E-02 |
| LOC100537991 | -1.34 | 3.56E-06 | 3.57E-03 |  | dap1b | 0.63 | 8.45E-05 | 8.46E-02 |
| gngt1 | 3.33 | 4.04E-06 | 3.94E-03 |  | LOC101884422 | -7.97 | 8.91E-05 | 8.53E-02 |
| hsd17b7 | 1.18 | 4.28E-06 | 4.06E-03 |  | sagb | -7.46 | 8.97E-05 | 8.53E-02 |
| mid1ip1b | 0.88 | 4.37E-06 | 4.06E-03 |  | pank1b | -0.76 | 1.04E-04 | 9.70E-02 |
| ercc6l | 1.51 | 4.77E-06 | 4.32E-03 |  | pcdh1a3 | -3.13 | 1.42E-04 | 1.29E-01 |
| mvda | 1.49 | 6.43E-06 | 5.69E-03 |  | si:dkey-188i13.10 | -1.17 | 1.51E-04 | 1.33E-01 |
| atp2a1 | -4.91 | 6.67E-06 | 5.74E-03 |  | LOC101883296 | -4.34 | 1.56E-04 | 1.35E-01 |
| zgc:194125 | 8.99 | 6.79E-06 | 5.74E-03 |  | zgc:162267 | -7.58 | 1.71E-04 | 1.44E-01 |
| pdk4 | -1.72 | 8.50E-06 | 7.03E-03 |  | si:ch211-5k11.6 | -2.11 | 1.78E-04 | 1.44E-01 |
| dbi | 0.67 | 9.08E-06 | 7.35E-03 |  | igfn1.2 | -7.37 | 1.78E-04 | 1.44E-01 |
| sh2d3cb | -0.82 | 9.31E-06 | 7.38E-03 |  | pcdh2ab5 | -3.39 | 2.11E-04 | 1.62E-01 |
| hsd17b3 | -1.14 | 1.09E-05 | 8.48E-03 |  | smtlb | -1.67 | 2.12E-04 | 1.62E-01 |
| slc43a2a | -1.15 | 1.16E-05 | 8.80E-03 |  | fdps | -0.78 | 2.15E-04 | 1.62E-01 |
| mcm5 | 1.62 | 1.28E-05 | 9.55E-03 |  | bmp2k | -0.60 | 2.17E-04 | 1.62E-01 |
| hspa4a | 0.91 | 1.50E-05 | 1.09E-02 |  | LOC100537796 | -4.98 | 2.33E-04 | 1.70E-01 |
| slc39a10 | 0.87 | 1.54E-05 | 1.09E-02 |  | si:ch73-52e5.2 | -0.84 | 2.38E-04 | 1.70E-01 |
| LOC101882848 | -4.25 | 1.55E-05 | 1.09E-02 |  | zgc:109965 | -7.07 | 2.42E-04 | 1.70E-01 |
| rcn3 | 1.87 | 1.65E-05 | 1.13E-02 |  | LOC100330779 | -2.62 | 2.50E-04 | 1.73E-01 |
| chaf1b | 1.50 | 1.66E-05 | 1.13E-02 |  | hmgcra | -0.89 | 2.76E-04 | 1.86E-01 |
| galn | -0.88 | 2.02E-05 | 1.35E-02 |  | ugt5g1 | -1.28 | 2.79E-04 | 1.86E-01 |
| LOC101882585 | -1.01 | 2.15E-05 | 1.40E-02 |  | fbn2b-2 | -7.67 | 3.25E-04 | 2.13E-01 |
| tcirg1 | -0.98 | 2.18E-05 | 1.40E-02 |  | LOC103909359 | -1.47 | 3.45E-04 | 2.22E-01 |
| wfikkn2 | 0.82 | 2.37E-05 | 1.48E-02 |  | sqlea | -0.92 | 3.57E-04 | 2.26E-01 |
| asmt | 2.94 | 2.38E-05 | 1.48E-02 |  | atp2a1 | 2.14 | 3.66E-04 | 2.28E-01 |
| trip13 | 2.31 | 2.58E-05 | 1.57E-02 |  | si:dkey-261e22.1 | -0.62 | 3.83E-04 | 2.32E-01 |
| glmnb | 5.38 | 2.63E-05 | 1.57E-02 |  | wwp1 | -9.18 | 3.88E-04 | 2.32E-01 |
| prkacbb | -11.35 | 2.64E-05 | 1.57E-02 |  | ugt1b7 | -4.18 | 3.91E-04 | 2.32E-01 |
| arr3a | 3.59 | 2.85E-05 | 1.67E-02 |  | idi1 | -0.75 | 4.14E-04 | 2.42E-01 |
| prnpb | 1.23 | 3.02E-05 | 1.74E-02 |  | insig1 | -0.96 | 4.23E-04 | 2.42E-01 |
| cbx7a | -0.77 | 3.37E-05 | 1.91E-02 |  | zgc:175214 | 0.53 | 4.26E-04 | 2.42E-01 |
| mybl2b | 1.21 | 3.54E-05 | 1.98E-02 |  | fbn2b | -1.56 | 4.33E-04 | 2.42E-01 |
| pole | 1.30 | 3.90E-05 | 2.15E-02 |  |  |  |  |  |
| sfrp5 | 0.86 | 4.26E-05 | 2.29E-02 |  |  |  |  |  |
| prl2 | 1.18 | 4.28E-05 | 2.29E-02 |  |  |  |  |  |
| zgc:194659 | -1.20 | 4.59E-05 | 2.40E-02 |  |  |  |  |  |
| tmem97 | 0.99 | 4.61E-05 | 2.40E-02 |  |  |  |  |  |
| LOC101883296 | 4.24 | 4.78E-05 | 2.46E-02 |  |  |  |  |  |
| igfn1.3 | -7.64 | 4.90E-05 | 2.48E-02 |  |  |  |  |  |
| pla2g4ab | -7.43 | 5.02E-05 | 2.51E-02 |  |  |  |  |  |
| mcm4 | 1.28 | 5.08E-05 | 2.51E-02 |  |  |  |  |  |
| aqp9b | 1.39 | 5.86E-05 | 2.81E-02 |  |  |  |  |  |
| steap4 | 0.70 | 5.87E-05 | 2.81E-02 |  |  |  |  |  |
| pmelb | 1.22 | 5.92E-05 | 2.81E-02 |  |  |  |  |  |
| tefa | -0.62 | 6.03E-05 | 2.83E-02 |  |  |  |  |  |
| mcm6 | 1.28 | 6.65E-05 | 3.09E-02 |  |  |  |  |  |
| hbp1 | -1.40 | 6.74E-05 | 3.09E-02 |  |  |  |  |  |
| si:dkey-22i16.2 | 5.18 | 6.83E-05 | 3.10E-02 |  |  |  |  |  |
| elna | 0.92 | 8.16E-05 | 3.65E-02 |  |  |  |  |  |
| ccbl2 | -8.32 | 8.59E-05 | 3.80E-02 |  |  |  |  |  |
| acat2 | 0.72 | 8.81E-05 | 3.85E-02 |  |  |  |  |  |
| LOC100537980 | 8.11 | 8.97E-05 | 3.85E-02 |  |  |  |  |  |
| lect2l | 2.38 | 9.01E-05 | 3.85E-02 |  |  |  |  |  |
| si:ch211-245j22.3 | 3.14 | 9.14E-05 | 3.86E-02 |  |  |  |  |  |
| pcmtd2 | -0.81 | 9.42E-05 | 3.94E-02 |  |  |  |  |  |
| pi4k2a | -1.47 | 9.68E-05 | 4.01E-02 |  |  |  |  |  |
| pcsk9 | 1.14 | 1.00E-04 | 4.10E-02 |  |  |  |  |  |
| ajuba | 1.09 | 1.03E-04 | 4.17E-02 |  |  |  |  |  |
| rbp4l | 2.86 | 1.15E-04 | 4.59E-02 |  |  |  |  |  |
| pcmtl | -0.77 | 1.18E-04 | 4.68E-02 |  |  |  |  |  |
| saga | 3.62 | 1.28E-04 | 5.04E-02 |  |  |  |  |  |
| dnmt4 | 0.95 | 1.41E-04 | 5.47E-02 |  |  |  |  |  |
| pcdh1a3 | 2.62 | 1.56E-04 | 6.00E-02 |  |  |  |  |  |
| LOC101883763 | 7.83 | 1.61E-04 | 6.10E-02 |  |  |  |  |  |
| mcm2 | 1.40 | 1.63E-04 | 6.10E-02 |  |  |  |  |  |
| chaf1a | 0.94 | 1.64E-04 | 6.10E-02 |  |  |  |  |  |
| scarb2 | 0.71 | 1.98E-04 | 7.32E-02 |  |  |  |  |  |
| asf1ba | 1.33 | 2.15E-04 | 7.78E-02 |  |  |  |  |  |
| h1fx | -1.17 | 2.16E-04 | 7.78E-02 |  |  |  |  |  |
| pcna | 0.82 | 2.18E-04 | 7.78E-02 |  |  |  |  |  |
| zgc:114181 | 0.65 | 2.21E-04 | 7.78E-02 |  |  |  |  |  |
| srsf5a | -0.75 | 2.21E-04 | 7.78E-02 |  |  |  |  |  |
| col18a1-2 | 0.74 | 2.27E-04 | 7.93E-02 |  |  |  |  |  |
| prim1 | 1.06 | 2.70E-04 | 9.32E-02 |  |  |  |  |  |
| serpinh1a | 1.14 | 2.79E-04 | 9.56E-02 |  |  |  |  |  |
| zgc:172244 | 2.04 | 2.85E-04 | 9.70E-02 |  |  |  |  |  |
| LOC792903 | 1.62 | 3.07E-04 | 1.03E-01 |  |  |  |  |  |
| LOC103909872 | 9.47 | 3.11E-04 | 1.03E-01 |  |  |  |  |  |
| trnC | -0.71 | 3.12E-04 | 1.03E-01 |  |  |  |  |  |
| mgea5 | -0.80 | 3.14E-04 | 1.03E-01 |  |  |  |  |  |
| gins2 | 1.59 | 3.19E-04 | 1.04E-01 |  |  |  |  |  |
| tfr1a | 1.31 | 3.25E-04 | 1.05E-01 |  |  |  |  |  |
| slc16a12a | -1.44 | 3.40E-04 | 1.09E-01 |  |  |  |  |  |
| abcg4b | 1.72 | 3.48E-04 | 1.09E-01 |  |  |  |  |  |
| tk1 | 1.09 | 3.50E-04 | 1.09E-01 |  |  |  |  |  |
| bnip3 | -0.57 | 3.54E-04 | 1.09E-01 |  |  |  |  |  |
| htr3b | 7.28 | 3.55E-04 | 1.09E-01 |  |  |  |  |  |
| mcm3 | 0.99 | 3.59E-04 | 1.09E-01 |  |  |  |  |  |
| zmp:0000001082 | -0.63 | 3.60E-04 | 1.09E-01 |  |  |  |  |  |
| LOC101884924 | -1.37 | 3.67E-04 | 1.11E-01 |  |  |  |  |  |
| pth2 | -0.84 | 3.74E-04 | 1.11E-01 |  |  |  |  |  |
| LOC567822 | 2.64 | 3.74E-04 | 1.11E-01 |  |  |  |  |  |
| pcdh1g18 | -1.11 | 3.76E-04 | 1.11E-01 |  |  |  |  |  |
| si:busm1-48c11.3 | 1.88 | 3.92E-04 | 1.15E-01 |  |  |  |  |  |
| pola2 | 0.88 | 4.03E-04 | 1.17E-01 |  |  |  |  |  |
| LOC100537554 | 0.66 | 4.09E-04 | 1.17E-01 |  |  |  |  |  |
| asxl1 | -1.17 | 4.10E-04 | 1.17E-01 |  |  |  |  |  |
| dscc1 | 1.82 | 4.13E-04 | 1.17E-01 |  |  |  |  |  |
| pold1 | 0.88 | 4.15E-04 | 1.17E-01 |  |  |  |  |  |
| fosb | -1.54 | 4.24E-04 | 1.19E-01 |  |  |  |  |  |
| wdhd1 | 1.39 | 4.35E-04 | 1.21E-01 |  |  |  |  |  |
| neflb | 0.51 | 4.52E-04 | 1.24E-01 |  |  |  |  |  |
| znf367 | 1.22 | 4.53E-04 | 1.24E-01 |  |  |  |  |  |
| rbm4.1 | 0.73 | 4.70E-04 | 1.28E-01 |  |  |  |  |  |
| zgc:56306 | -0.87 | 4.82E-04 | 1.30E-01 |  |  |  |  |  |
| scd | 0.70 | 4.84E-04 | 1.30E-01 |  |  |  |  |  |
| zgc:103625 | 5.33 | 4.91E-04 | 1.31E-01 |  |  |  |  |  |
| LOC100537796 | 3.01 | 5.04E-04 | 1.33E-01 |  |  |  |  |  |
| igfbp1a | -1.02 | 5.06E-04 | 1.33E-01 |  |  |  |  |  |
| mvk | 1.16 | 5.61E-04 | 1.46E-01 |  |  |  |  |  |
| pigp | 0.85 | 5.65E-04 | 1.46E-01 |  |  |  |  |  |
| LOC100535624 | 1.96 | 5.76E-04 | 1.48E-01 |  |  |  |  |  |
| pcolcea | 0.62 | 5.80E-04 | 1.48E-01 |  |  |  |  |  |
| zgc:171775 | -1.97 | 5.82E-04 | 1.48E-01 |  |  |  |  |  |
| slc1a5 | 0.98 | 5.88E-04 | 1.48E-01 |  |  |  |  |  |
| rrm1 | 0.82 | 5.96E-04 | 1.49E-01 |  |  |  |  |  |
| tubb5 | 0.65 | 6.02E-04 | 1.50E-01 |  |  |  |  |  |
| srsf5b | -0.83 | 6.08E-04 | 1.50E-01 |  |  |  |  |  |
| cenph | 1.77 | 6.09E-04 | 1.50E-01 |  |  |  |  |  |
| rnpep | 0.84 | 6.22E-04 | 1.51E-01 |  |  |  |  |  |
| bbox1 | -0.98 | 6.22E-04 | 1.51E-01 |  |  |  |  |  |
| nutf2l | 1.08 | 6.31E-04 | 1.52E-01 |  |  |  |  |  |
| LOC103909793 | -8.01 | 6.49E-04 | 1.55E-01 |  |  |  |  |  |
| slc16a9b | -0.97 | 6.73E-04 | 1.60E-01 |  |  |  |  |  |
| gpcpd1 | -0.60 | 6.90E-04 | 1.62E-01 |  |  |  |  |  |
| zgc:110540 | 1.08 | 6.91E-04 | 1.62E-01 |  |  |  |  |  |
| lpin1 | -1.35 | 7.07E-04 | 1.63E-01 |  |  |  |  |  |
| znf318 | 0.55 | 7.07E-04 | 1.63E-01 |  |  |  |  |  |
| elovl2 | 0.74 | 7.08E-04 | 1.63E-01 |  |  |  |  |  |
| dtl | 1.82 | 7.23E-04 | 1.63E-01 |  |  |  |  |  |
| tyms | 1.35 | 7.25E-04 | 1.63E-01 |  |  |  |  |  |
| rdh10a | 0.60 | 7.25E-04 | 1.63E-01 |  |  |  |  |  |
| srsf11 | 0.69 | 7.27E-04 | 1.63E-01 |  |  |  |  |  |
| tcp11l2 | -0.80 | 7.30E-04 | 1.63E-01 |  |  |  |  |  |
| LOC101886910 | 8.74 | 7.43E-04 | 1.63E-01 |  |  |  |  |  |
| si:dkey-5n18.1 | 1.12 | 7.44E-04 | 1.63E-01 |  |  |  |  |  |
| si:busm1-194e12.12 | 1.68 | 7.46E-04 | 1.63E-01 |  |  |  |  |  |
| rtcb | 0.57 | 7.47E-04 | 1.63E-01 |  |  |  |  |  |
| hells | 0.93 | 7.61E-04 | 1.65E-01 |  |  |  |  |  |
| tm7sf2 | 0.82 | 7.65E-04 | 1.65E-01 |  |  |  |  |  |
| hsd17b12a | -0.88 | 7.80E-04 | 1.67E-01 |  |  |  |  |  |
| wfdc1 | 1.75 | 7.80E-04 | 1.67E-01 |  |  |  |  |  |
| rbp5 | 0.69 | 8.08E-04 | 1.71E-01 |  |  |  |  |  |
| ccdc79 | 1.08 | 8.10E-04 | 1.71E-01 |  |  |  |  |  |
| pik3ip1 | -0.61 | 8.35E-04 | 1.75E-01 |  |  |  |  |  |
| tanc2a | -0.52 | 8.48E-04 | 1.77E-01 |  |  |  |  |  |
| fes | -0.95 | 8.60E-04 | 1.79E-01 |  |  |  |  |  |
| crabp2a | 0.95 | 8.71E-04 | 1.79E-01 |  |  |  |  |  |
| thbs4a | -1.19 | 8.74E-04 | 1.79E-01 |  |  |  |  |  |
| e2f8 | 1.17 | 8.77E-04 | 1.79E-01 |  |  |  |  |  |
| fkbp14 | 1.03 | 9.07E-04 | 1.84E-01 |  |  |  |  |  |
| rag1 | -3.20 | 9.24E-04 | 1.87E-01 |  |  |  |  |  |
| atg9a | -0.55 | 9.61E-04 | 1.93E-01 |  |  |  |  |  |
| cyp19a1b | -0.93 | 9.82E-04 | 1.97E-01 |  |  |  |  |  |
| LOC100332293 | -0.81 | 1.01E-03 | 2.01E-01 |  |  |  |  |  |
| pde6g | 1.47 | 1.04E-03 | 2.06E-01 |  |  |  |  |  |
| si:dkey-27n6.1 | 7.80 | 1.05E-03 | 2.06E-01 |  |  |  |  |  |
| f13a1b | 0.88 | 1.06E-03 | 2.06E-01 |  |  |  |  |  |
| fads2 | 0.79 | 1.06E-03 | 2.06E-01 |  |  |  |  |  |
| LOC100537097 | -7.12 | 1.07E-03 | 2.06E-01 |  |  |  |  |  |
| si:ch211-80h18.1 | 0.54 | 1.07E-03 | 2.06E-01 |  |  |  |  |  |
| si:ch211-236l14.4 | 0.79 | 1.07E-03 | 2.06E-01 |  |  |  |  |  |
| zmp:0000000612 | 3.59 | 1.08E-03 | 2.06E-01 |  |  |  |  |  |
| crybb2 | 2.03 | 1.09E-03 | 2.08E-01 |  |  |  |  |  |
| ncaph2 | 1.00 | 1.11E-03 | 2.10E-01 |  |  |  |  |  |
| col11a1b | 1.16 | 1.11E-03 | 2.10E-01 |  |  |  |  |  |
| lipg | 0.74 | 1.14E-03 | 2.14E-01 |  |  |  |  |  |
| LOC103911187 | 8.55 | 1.15E-03 | 2.14E-01 |  |  |  |  |  |
| eif4ebp3l | -1.23 | 1.16E-03 | 2.16E-01 |  |  |  |  |  |
| mb | -1.41 | 1.18E-03 | 2.18E-01 |  |  |  |  |  |
| LOC103909261 | -7.64 | 1.21E-03 | 2.21E-01 |  |  |  |  |  |
| noxo1b | -1.27 | 1.21E-03 | 2.21E-01 |  |  |  |  |  |
| top2a | 0.86 | 1.22E-03 | 2.21E-01 |  |  |  |  |  |
| timd4 | -0.73 | 1.23E-03 | 2.23E-01 |  |  |  |  |  |
| haus3 | 1.21 | 1.26E-03 | 2.25E-01 |  |  |  |  |  |
| dhcr7 | 0.62 | 1.26E-03 | 2.25E-01 |  |  |  |  |  |
| fabp11a | 0.61 | 1.27E-03 | 2.25E-01 |  |  |  |  |  |
| fkbp9 | 0.83 | 1.27E-03 | 2.25E-01 |  |  |  |  |  |
| lrrc8db | -1.06 | 1.27E-03 | 2.25E-01 |  |  |  |  |  |
| ccdc125 | -1.09 | 1.30E-03 | 2.28E-01 |  |  |  |  |  |
| tpc3 | 0.73 | 1.33E-03 | 2.33E-01 |  |  |  |  |  |
| cdca7a | 1.24 | 1.42E-03 | 2.47E-01 |  |  |  |  |  |
| ehd1b | 0.72 | 1.43E-03 | 2.48E-01 |  |  |  |  |  |
| gpr133 | 1.20 | 1.44E-03 | 2.49E-01 |  |  |  |  |  |
| LOC561086 | 0.83 | 1.44E-03 | 2.49E-01 |  |  |  |  |  |
| LOC794549 | 7.71 | 1.45E-03 | 2.49E-01 |  |  |  |  |  |
| rtn4rl2a | -0.99 | 1.46E-03 | 2.49E-01 |  |  |  |  |  |

List of DEGs with FDR of less than 0.25 and |fold change| above 1.2. FC, Fold Change; FDR, false discovery rate.
