# Supplementary material for: Different effects of methylphenidate and atomoxetine on the behavior and brain transcriptome of zebrafish
Source: Mol Brain. 2020 May 6;13:70. doi: 10.1186/s13041-020-00614-4 (PMC7203832; doi:10.1186/s13041-020-00614-4)
Supplement: Supplementary file 2 — Additional file 2: Table S2. KEGG pathway analysis for 8-day exposure. [file 13041_2020_614_MOESM2_ESM.docx]

**Table S2. KEGG pathway analysis for 8-day exposure.**

1) DEGs for 8-day MPH treatment.

| Term | P-value | Adjusted P-value |
| --- | --- | --- |
| Steroid biosynthesis | 2.15E-15 | 1.85E-13 |
| DNA replication | 9.13E-12 | 3.93E-10 |
| Terpenoid backbone biosynthesis | 2.98E-09 | 8.53E-08 |
| Biosynthesis of unsaturated fatty acids | 2.92E-04 | 6.28E-03 |
| Pyrimidine metabolism | 6.02E-04 | 1.04E-02 |
| Steroid hormone biosynthesis | 7.57E-04 | 1.09E-02 |
| Butanoate metabolism | 2.36E-03 | 2.90E-02 |
| Synthesis and degradation of ketone bodies | 4.27E-03 | 4.59E-02 |
| Cell cycle | 4.82E-03 | 4.61E-02 |
| Base excision repair | 8.14E-03 | 6.36E-02 |
| Phototransduction | 8.14E-03 | 6.36E-02 |
| Nucleotide excision repair | 1.31E-02 | 9.39E-02 |
| alpha-Linolenic acid metabolism | 1.70E-02 | 1.12E-01 |
| Tryptophan metabolism | 1.85E-02 | 1.13E-01 |
| Valine, leucine and isoleucine degradation | 2.37E-02 | 1.34E-01 |
| Mismatch repair | 2.49E-02 | 1.34E-01 |
| PPAR signaling pathway | 3.79E-02 | 1.92E-01 |

2) DEGs for 8-day ATX treatment.

| Term | P-value | Adjusted P-value |
| --- | --- | --- |
| Terpenoid backbone biosynthesis | 8.54E-07 | 2.22E-05 |
| Steroid biosynthesis | 1.88E-03 | 2.44E-02 |
| Phototransduction | 7.03E-03 | 5.31E-02 |
| Retinol metabolism | 8.17E-03 | 5.31E-02 |
| Synthesis and degradation of ketone bodies | 3.02E-02 | 1.57E-01 |
| Pantothenate and CoA biosynthesis | 4.33E-02 | 1.81E-01 |

3) Common DEGs for 8-day MPH and ATX treatment.

| Term | P-value | Adjusted P-value |
| --- | --- | --- |
| Terpenoid backbone biosynthesis | 3.32E-09 | 3.66E-08 |
| Steroid biosynthesis | 1.30E-04 | 7.13E-04 |
| Synthesis and degradation of ketone bodies | 8.07E-03 | 2.96E-02 |
| Butanoate metabolism | 2.14E-02 | 5.88E-02 |
| Phototransduction | 3.28E-02 | 7.21E-02 |
| Tryptophan metabolism | 4.41E-02 | 7.24E-02 |
| Valine, leucine and isoleucine degradation | 4.84E-02 | 7.24E-02 |
