# Supplementary material for: Different effects of methylphenidate and atomoxetine on the behavior and brain transcriptome of zebrafish
Source: Mol Brain. 2020 May 6;13:70. doi: 10.1186/s13041-020-00614-4 (PMC7203832; doi:10.1186/s13041-020-00614-4)
Supplement: Supplementary file 3 — Additional file 3: Table S3. Differential expression profiles for 4-h exposure. [file 13041_2020_614_MOESM3_ESM.docx]

**Table S3.** **Differential expression profiles for 4-hour exposure.**

| **MPH for 4 hours** |  |  |  |  | **ATX for 4 hours** |  |  |  |
| --- | --- | --- | --- | --- | --- | --- | --- | --- |
| **Gene Symbol** | **logFC** | **P-value** | **FDR** |  | **Gene Symbol** | **logFC** | **P-value** | **FDR** |
| aldh9a1a.2 | -10.17 | 9.85E-14 | 3.75E-09 |  | zgc:103625 | -7.05 | 1.88E-57 | 7.17E-53 |
| mylpfa | -3.31 | 3.71E-12 | 7.06E-08 |  | si:ch73-199e17.1 | 6.14 | 3.31E-37 | 6.29E-33 |
| wwp1 | -10.46 | 9.38E-12 | 1.19E-07 |  | pcdh2ab12 | 3.65 | 2.76E-34 | 3.50E-30 |
| MGC174152 | -9.87 | 2.40E-11 | 2.29E-07 |  | f13a1a.1 | -3.59 | 3.22E-21 | 3.06E-17 |
| trpa1a | -1.66 | 8.67E-11 | 5.80E-07 |  | pcdh2g29-2 | -2.63 | 6.20E-16 | 4.72E-12 |
| actc1b | -2.19 | 9.14E-11 | 5.80E-07 |  | LOC101883687 | 9.52 | 2.66E-15 | 1.68E-11 |
| pvalb4 | -3.10 | 2.01E-09 | 1.09E-05 |  | pkmb | -9.51 | 1.00E-12 | 5.45E-09 |
| wu:fb36g12 | -9.26 | 3.08E-09 | 1.46E-05 |  | LOC100004091 | 3.64 | 1.74E-12 | 8.29E-09 |
| si:dkey-26g8.4 | -5.82 | 2.50E-08 | 1.06E-04 |  | ckmb | -4.06 | 6.93E-12 | 2.93E-08 |
| LOC101886687 | 9.27 | 2.97E-08 | 1.13E-04 |  | pcdh2g28-2 | 2.24 | 9.41E-12 | 3.58E-08 |
| pcdh1gc6 | -1.00 | 3.70E-08 | 1.28E-04 |  | si:ch211-194e18.2 | 1.14 | 1.73E-11 | 6.00E-08 |
| ckmt2a | -8.29 | 4.84E-08 | 1.53E-04 |  | pcdh10a | -0.91 | 2.65E-11 | 8.41E-08 |
| pvalb2 | -3.46 | 5.85E-08 | 1.71E-04 |  | pygma | -4.15 | 7.23E-10 | 2.12E-06 |
| pcdh2ab9 | -1.66 | 8.68E-08 | 2.36E-04 |  | LOC103909396 | -8.63 | 2.77E-09 | 7.33E-06 |
| mybpha | -5.55 | 9.53E-08 | 2.42E-04 |  | LOC101884148 | 8.46 | 2.89E-09 | 7.33E-06 |
| LOC100007086 | -3.40 | 1.40E-07 | 3.32E-04 |  | si:ch211-244b2.1 | 0.93 | 1.41E-08 | 3.35E-05 |
| atp2a1 | -6.08 | 1.57E-07 | 3.51E-04 |  | ckma | -2.88 | 1.79E-08 | 4.00E-05 |
| si:ch73-236c18.7 | 4.46 | 1.90E-07 | 4.02E-04 |  | atp2a1 | -3.63 | 1.94E-07 | 4.09E-04 |
| pcdh1a4 | -3.57 | 3.37E-07 | 6.75E-04 |  | c5h11orf1 | -1.83 | 3.15E-07 | 6.31E-04 |
| ckma | -2.76 | 4.12E-07 | 7.84E-04 |  | LOC100007086 | -5.55 | 4.47E-07 | 8.50E-04 |
| tnnt3b | -2.46 | 9.11E-07 | 1.65E-03 |  | LOC103911039 | -8.83 | 6.82E-07 | 1.24E-03 |
| LOC103909877 | 8.89 | 1.10E-06 | 1.91E-03 |  | gapdh | -1.33 | 7.50E-07 | 1.30E-03 |
| LOC101884724 | 9.92 | 1.18E-06 | 1.96E-03 |  | spp1 | -1.92 | 1.03E-06 | 1.71E-03 |
| lgals3bpa | 9.42 | 1.54E-06 | 2.43E-03 |  | LOC103909359 | -2.02 | 1.49E-06 | 2.37E-03 |
| LOC100149540 | -8.22 | 1.69E-06 | 2.57E-03 |  | socs3a | -2.07 | 1.78E-06 | 2.63E-03 |
| ckmb | -3.46 | 1.76E-06 | 2.57E-03 |  | pvalb4 | -3.34 | 1.82E-06 | 2.63E-03 |
| zgc:175128 | -1.36 | 1.98E-06 | 2.79E-03 |  | zgc:55943 | -1.18 | 1.87E-06 | 2.63E-03 |
| nme2b.2 | -1.36 | 4.14E-06 | 5.62E-03 |  | mylpfa | -3.85 | 2.01E-06 | 2.74E-03 |
| mylz3 | -1.76 | 1.04E-05 | 1.37E-02 |  | tnni2b.1 | -7.70 | 2.55E-06 | 3.24E-03 |
| pcdh10a | 1.12 | 1.31E-05 | 1.66E-02 |  | zgc:173425 | -7.80 | 2.56E-06 | 3.24E-03 |
| LOC101882848 | -4.30 | 1.39E-05 | 1.70E-02 |  | atp2a1l | -5.25 | 4.33E-06 | 5.32E-03 |
| atp2a1l | -2.86 | 1.44E-05 | 1.71E-02 |  | ntn1b | 0.73 | 6.85E-06 | 7.96E-03 |
| LOC101883763 | 8.64 | 3.84E-05 | 4.43E-02 |  | slc25a18 | -0.95 | 6.91E-06 | 7.96E-03 |
| ier2 | -1.13 | 4.69E-05 | 5.25E-02 |  | actc1b | -2.92 | 8.15E-06 | 9.12E-03 |
| tnni2b.2 | -1.84 | 6.71E-05 | 6.99E-02 |  | ldb3b | -3.33 | 1.04E-05 | 1.11E-02 |
| tnnc2 | -1.47 | 6.76E-05 | 6.99E-02 |  | tnni2b.2 | -3.87 | 1.05E-05 | 1.11E-02 |
| LOC103908995 | -8.55 | 6.91E-05 | 6.99E-02 |  | tnnt3b | -3.07 | 1.33E-05 | 1.37E-02 |
| zgc:174855 | -2.74 | 6.98E-05 | 6.99E-02 |  | LOC572412 | -7.49 | 1.50E-05 | 1.51E-02 |
| grk7b | -7.12 | 8.73E-05 | 8.38E-02 |  | rpp25 | -3.27 | 1.55E-05 | 1.51E-02 |
| arrdc3b | -1.43 | 8.81E-05 | 8.38E-02 |  | LOC103909127 | -7.53 | 1.61E-05 | 1.53E-02 |
| ccbl2 | -8.32 | 9.13E-05 | 8.47E-02 |  | isg15 | 1.63 | 2.00E-05 | 1.86E-02 |
| gapdh | -1.72 | 1.01E-04 | 9.18E-02 |  | LOC103909728 | -7.37 | 2.24E-05 | 2.03E-02 |
| junbb | -2.56 | 1.06E-04 | 9.39E-02 |  | mmp13b | -3.21 | 2.72E-05 | 2.40E-02 |
| mrps18c | -0.90 | 1.23E-04 | 1.06E-01 |  | si:dkey-26g8.4 | -7.89 | 4.09E-05 | 3.54E-02 |
| htr3b | 7.85 | 1.26E-04 | 1.06E-01 |  | myhb | -10.12 | 4.18E-05 | 3.54E-02 |
| henmt1 | 2.60 | 1.38E-04 | 1.14E-01 |  | si:ch1073-358c10.1 | -1.53 | 4.71E-05 | 3.90E-02 |
| LOC100536103 | 3.14 | 1.45E-04 | 1.15E-01 |  | myha | -5.99 | 6.24E-05 | 5.05E-02 |
| fos | -1.26 | 1.45E-04 | 1.15E-01 |  | pou2f2a | -0.89 | 7.03E-05 | 5.57E-02 |
| LOC103908788 | -3.45 | 1.55E-04 | 1.19E-01 |  | abcb5 | -2.62 | 8.07E-05 | 6.20E-02 |
| myhz1.1 | -5.19 | 1.56E-04 | 1.19E-01 |  | paqr3b | -5.00 | 8.15E-05 | 6.20E-02 |
| zgc:171704 | -2.86 | 1.60E-04 | 1.20E-01 |  | ttnb | -1.60 | 1.00E-04 | 7.46E-02 |
| mb | -1.65 | 1.66E-04 | 1.21E-01 |  | nme2b.2 | -2.00 | 1.05E-04 | 7.71E-02 |
| LOC101884483 | -9.95 | 2.04E-04 | 1.46E-01 |  | gh1 | 9.37 | 1.09E-04 | 7.81E-02 |
| LOC103909882 | -3.44 | 2.07E-04 | 1.46E-01 |  | snap91 | -0.56 | 1.18E-04 | 8.31E-02 |
| LOC101883170 | 7.60 | 2.11E-04 | 1.46E-01 |  | bzrap1 | -7.07 | 1.26E-04 | 8.73E-02 |
| actn3a | -1.54 | 2.22E-04 | 1.51E-01 |  | ubac1 | 0.76 | 1.29E-04 | 8.73E-02 |
| LOC103911801 | -8.22 | 2.54E-04 | 1.69E-01 |  | rn7sk | 1.11 | 1.61E-04 | 1.06E-01 |
| lim2.3 | -8.52 | 2.98E-04 | 1.94E-01 |  | fgfbp1 | 0.73 | 1.61E-04 | 1.06E-01 |
| pcdh2ab10 | 1.73 | 3.01E-04 | 1.94E-01 |  | chka | -0.56 | 2.07E-04 | 1.32E-01 |
| pcdh2ab8 | 1.99 | 3.08E-04 | 1.96E-01 |  | LOC101886687 | -3.00 | 2.09E-04 | 1.32E-01 |
| zgc:194125 | 7.09 | 3.21E-04 | 2.00E-01 |  | henmt1 | -3.33 | 2.78E-04 | 1.68E-01 |
| urah | 7.58 | 3.35E-04 | 2.06E-01 |  | mylz3 | -2.33 | 2.80E-04 | 1.68E-01 |
| tnni2a.3 | -3.45 | 3.47E-04 | 2.08E-01 |  | mc5ra | 2.22 | 2.82E-04 | 1.68E-01 |
| ckmt2b | -3.74 | -3.74 | 3.49E-04 |  | pvalb2 | -4.06 | 2.83E-04 | 1.68E-01 |
| wu:fa26c03 | -2.58 | -2.58 | 3.66E-04 |  | entpd5a | -2.08 | 3.02E-04 | 1.74E-01 |
| myoz1a | -6.97 | -6.97 | 4.04E-04 |  | LOC103911524 | 8.88 | 3.04E-04 | 1.74E-01 |
| pcdh1g31 | 0.69 | 0.69 | 4.18E-04 |  | icn | 0.62 | 3.06E-04 | 1.74E-01 |
| LOC103909872 | 9.16 | 9.16 | 4.33E-04 |  | aldh9a1a.2 | 9.04 | 3.13E-04 | 1.75E-01 |
| LOC100534757 | -9.21 | -9.21 | 4.37E-04 |  | kcnd1 | -0.78 | 3.21E-04 | 1.75E-01 |
|  |  |  |  |  | rsrp1 | -1.44 | 3.25E-04 | 1.75E-01 |
|  |  |  |  |  | anxa2a | 0.86 | 3.26E-04 | 1.75E-01 |
|  |  |  |  |  | myhc4 | -4.71 | 3.50E-04 | 1.83E-01 |
|  |  |  |  |  | LOC103910140 | 1.09 | 3.52E-04 | 1.83E-01 |
|  |  |  |  |  | cryabb | -1.17 | 3.85E-04 | 1.98E-01 |
|  |  |  |  |  | scn2b | -0.61 | 4.14E-04 | 2.10E-01 |
|  |  |  |  |  | si:dkey-8k3.2 | 0.73 | 4.29E-04 | 2.15E-01 |
|  |  |  |  |  | si:ch211-223m11.2 | -7.50 | 4.39E-04 | 2.17E-01 |
|  |  |  |  |  | si:ch211-281g13.4 | 8.09 | 4.88E-04 | 2.38E-01 |

List of DEGs with FDR of less than 0.25 and |fold change| above 1.2. FC, Fold Change; FDR, false discovery rate.
