# Supplementary material for: Different effects of methylphenidate and atomoxetine on the behavior and brain transcriptome of zebrafish
Source: Mol Brain. 2020 May 6;13:70. doi: 10.1186/s13041-020-00614-4 (PMC7203832; doi:10.1186/s13041-020-00614-4)
Supplement: Supplementary file 4 — Additional file 4: Table S4. KEGG pathway analysis for 4-h exposure. [file 13041_2020_614_MOESM4_ESM.docx]

**Table S4. KEGG pathway analysis for 4-hour exposure.**

1) DEGs for 4-hour MPH treatment.

| Term | P-value | Adjusted P-value |
| --- | --- | --- |
| Arginine and proline metabolism | 5.84E-05 | 9.92E-04 |
| Selenocompound metabolism | 0.05 | 0.37 |
| Calcium signaling pathway | 0.07 | 0.37 |
| Cysteine and methionine metabolism | 0.16 | 0.44 |
| Tryptophan metabolism | 0.16 | 0.44 |
| Phototransduction | 0.12 | 0.44 |
| Pyrimidine metabolism | 0.19 | 0.44 |
| Drug metabolism | 0.23 | 0.44 |
| Glycolysis / Gluconeogenesis | 0.23 | 0.44 |
| Cardiac muscle contraction | 0.29 | 0.50 |

2) DEGs for 4-hour ATX treatment.

| Term | P-value | Adjusted P-value |
| --- | --- | --- |
| Arginine and proline metabolism | 0.02 | 0.16 |
| Purine metabolism | 0.02 | 0.16 |
| Pyrimidine metabolism | 0.02 | 0.16 |
| Glycolysis / Gluconeogenesis | 0.04 | 0.18 |
| Starch and sucrose metabolism | 0.12 | 0.46 |
| ABC transporters | 0.14 | 0.46 |
| Pyruvate metabolism | 0.16 | 0.47 |
| Tight junction | 0.20 | 0.50 |
| Drug metabolism | 0.26 | 0.54 |
| Glycerophospholipid metabolism | 0.32 | 0.54 |

3) Common DEGs for 4-hour MPH and ATX treatment.

| Term | P-value | Adjusted P-value |
| --- | --- | --- |
| Arginine and proline metabolism | 1.51E-03 | 1.51E-02 |
| Calcium signaling pathway | 0.03 | 0.13 |
| Pyrimidine metabolism | 0.06 | 0.14 |
| Drug metabolism | 0.07 | 0.14 |
| Glycolysis / Gluconeogenesis | 0.07 | 0.14 |
| Cardiac muscle contraction | 0.09 | 0.15 |
| Purine metabolism | 0.13 | 0.19 |
| Adrenergic signaling in cardiomyocytes | 0.18 | 0.22 |
| Focal adhesion | 0.20 | 0.22 |
| Regulation of actin cytoskeleton | 0.22 | 0.22 |
