# Supplementary material for: Different effects of methylphenidate and atomoxetine on the behavior and brain transcriptome of zebrafish
Source: Mol Brain. 2020 May 6;13:70. doi: 10.1186/s13041-020-00614-4 (PMC7203832; doi:10.1186/s13041-020-00614-4)
Supplement: Supplementary file 5 — Additional file 5: Fig. S1. Dose-response curve of methylphenidate and atomoxetine. [file 13041_2020_614_MOESM5_ESM.docx]

**Figure S1.** **Dose-response curve of methylphenidate and atomoxetine.**

Time spent in top area and bottom area (y-axis) of the tank by fish treated with different doses of methylphenidate (MPH) or atomoxetine (ATX) (x-axis) are shown. The test was performed for the duration of 10 minutes (n=10 for each drug, n=12 for control). The dots represent the mean and the error bars stand for standard errors. **(a-b)**: The result of the novel tank test of MPH-treated fish for 4 hours. **a**: Time spent in top area (s). **b**: Time spent in bottom area (s). **(c-d)**: The result of the novel tank test of ATX-treated fish for 4 hours. **c**: Time spent in top area (s). **d**: Time spent in bottom area (s).

**
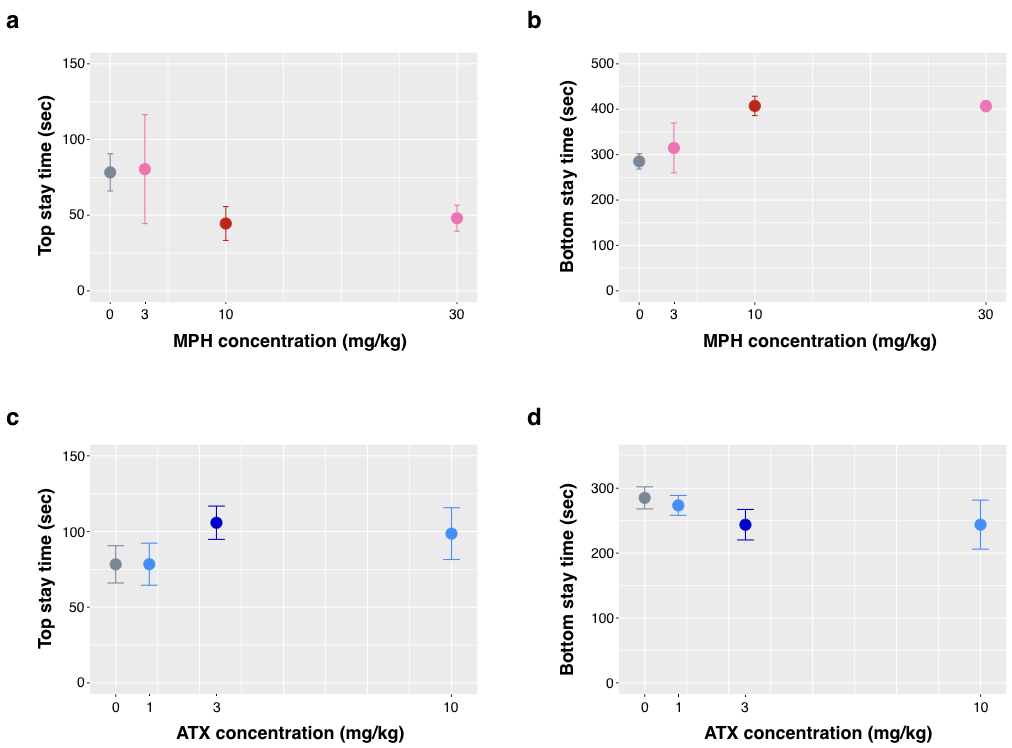
**
