# Supplementary material for: Different effects of methylphenidate and atomoxetine on the behavior and brain transcriptome of zebrafish
Source: Mol Brain. 2020 May 6;13:70. doi: 10.1186/s13041-020-00614-4 (PMC7203832; doi:10.1186/s13041-020-00614-4)
Supplement: Supplementary file 6 — Additional file 6: Fig. S2. The effect on behavior and transcriptome after 4 h of treatment of methylphenidate and atomoxetine. [file 13041_2020_614_MOESM6_ESM.docx]

**Figure S2.** **The effect on** **behavior and transcriptome after 4 hours of treatment of methylphenidate and atomoxetine.**

**(a-b)**: The results of the novel tank test of methylphenidate (MPH)- and atomoxetine (ATX)-treated fish for 4 hours. The test was performed in 10 fish per group for a duration of 10 minutes. **a**: Time spent in top area (s). **b:** Time spent in bottom area (s). Student's t-test was performed for the statistical analysis and asterisk denotes p<0.05. **(c-d)**: Differentially expressed genes (DEGs) in the brains of zebrafish treated with MPH or ATX. **c**: Venn diagram shows the total number of DEGs among MPH-treated and ATX-treated fish with respect to control. Analysis of differential expression in each cohort resulted in 70 DEGs between MPH-treated and controls, and 78 DEGs between ATX-treated and controls, 19 of which were shared between MPH-treated and ATX-treated fish. The DEGs were defined as genes with false discovery rate of less than 0.25 and absolute value of fold change larger than 1.2. **d**: Heatmap shows shared DEGs between MPH- and ATX-treated fish (rows) and drug exposure (columns). Blue represents down-regulated expression, red represents up-regulated expression, and white represents no change in expression.

**
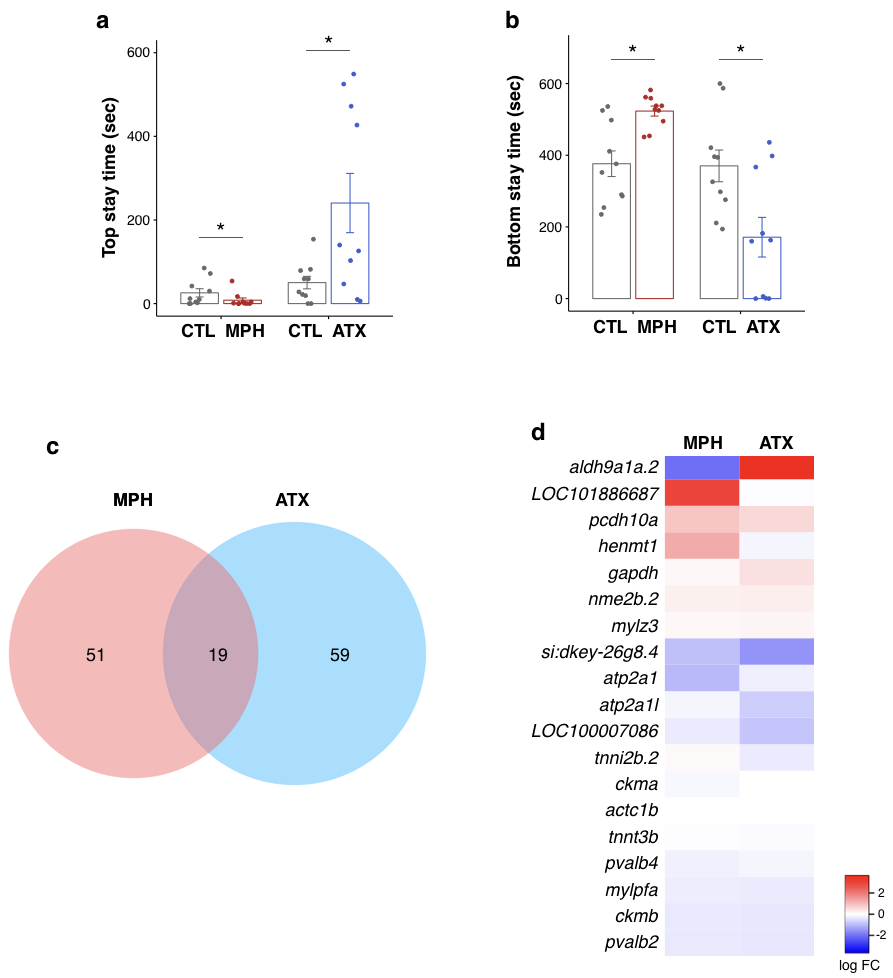
**
