# Supplementary material for: Different effects of methylphenidate and atomoxetine on the behavior and brain transcriptome of zebrafish
Source: Mol Brain. 2020 May 6;13:70. doi: 10.1186/s13041-020-00614-4 (PMC7203832; doi:10.1186/s13041-020-00614-4)
Supplement: Supplementary file 7 — Additional file 7: Materials and Methods [file 13041_2020_614_MOESM7_ESM.docx]

**Materials and Methods**

**Animals**

Adult zebrafish (*Danio rerio*) were housed in tanks with recirculating system under a 14 h/10 h light/dark cycle at 28℃. For dose determination, wild zebrafish were purchased (Meito Suien Co., Ltd., Nagoya, Japan) and used. AB wild-type strain fish were used for the behavior and transcriptome analyses. Fish were kept in groups of 3-11 individuals with a balanced sex ratio in tanks, and were fed once a day. For comparing control group with drug treated groups, fish with the same date of birth were equally distributed in each group. All zebrafish experiments were performed in accordance with the ethical guidelines of Kyoto University and were approved by the Institutional Animal Care and Use Committees of Kyoto University Graduate School of Medicine (approval number Med Kyo 19076).

**Dose determination and drug treatment**

To determine the appropriate dose of MPH and ATX, we conducted a novel tank assay using adult zebrafish. Methylphenidate hydrochloride (MPH; Sigma-Aldrich Japan, Tokyo, Japan) was dissolved in water at a concentration of 3, 10, and 30 mg/kg, while atomoxetine hydrochloride (ATX; Tokyo Chemical Industry Co., Tokyo, Japan) was dissolved in water at a concentration of 1, 3, and 10 mg/kg. Fish were treated for 4 hours in water containing the drug. The results of the novel tank test with different doses are shown in Additional file 5: Figure S1. The minimum dose that altered the behavior was adopted for further analysis using AB wild-type strain fish (10 mg/L for MPH, and 3 mg/L for ATX). The behavior and transcriptome analyses of AB wild-type strain fish were performed after 4-hour and 8-day drug exposure. For 8-day exposure, water containing the drug was replaced daily.

**Novel tank test**

The novel tank test was performed in a rectangular tank (width 200 mm, depth 50 mm, height 150 mm) filled with water to a height of 120 mm. The upper portion of the tank was defined as “top area” and the lower portion as “bottom area” (Fig. 1a). The test was performed according to previously described method [1]. Briefly, fish were individually introduced into the test tank, and their behaviors were recorded under 300 lux light for 10 minutes. Total distance traveled (mm), time spent in the top area (s), and time spent in the bottom area (s) were recorded. The test was performed in 10 fish per group. Data were analyzed using the Student’s *t*-test. Differences with a p-value of less than 0.05 were considered as statistically significant.

**Tissue collection and RNA isolation**

Following the drug treatment, whole brains of fish were removed as described previously [2]. Each brain was immediately immersed in RNAlater (Thermo Fisher Scientific, Waltham, MA, USA) and kept on ice. Total RNA was extracted from each brain using RNeasy Mini Kit (Qiagen, Hilden, Germany) according to the manufacturer's instructions. The quantity of RNA was measured using NanoDrop 2000 spectrophotometer (Thermo Fisher Scientific). The quality of RNA was assessed using the RNA 6000 Pico Kit (Agilent Technologies, Santa Clara, CA, USA) with Agilent 2100 Bioanalyzer. RNA samples with RNA integrity number larger than 8 were used for the following analysis.

**RNA-sequencing analysis**

In total, 26 RNA samples were isolated from three main groups: control (n = 10), MPH-treated (n = 8), and ATX-treated (n = 8). Each group was further divided into two subgroups (n = 4 or 5) for pooling samples for RNA-sequencing (RNA-seq) analysis. RNA-Seq libraries were prepared from 200 ng of total RNA using the TruSeq Stranded mRNA LT Sample Prep kit set A (Illumina, San Diego, CA, USA). Libraries were sequenced using Novaseq 6000 sequencing system (Illumina) with paired-end 101-bp reads. Sequencing and library preparation were performed by Macrogen Japan Corp. (Kyoto, Japan).

**Differential expression analysis**

The quality assessment of raw reads was performed with FastQC. Relevant adapter sequences were removed with Cutadapt. The reads were aligned against zebrafish reference genome (GRCz10) using Hisat2. The resulting BAM files were processed with SAMtools. The mapped reads were assembled into transcripts and calculated as gene expression level using StringTie. EdgeR in R was used to identify differentially expressed genes (DEGs). Genes with false discovery rate of less than 0.25 and absolute value of fold change larger than 1.2 were considered as DEGs.

**Pathway and network analysis**

Pathway analysis was performed using the Kyoto Encyclopedia of Genes and Genomes (KEGG) pathway database via FishEnrichr [3-5]. The enriched terms that had adjusted p-value less than 0.05 were considered as statistically significant. Network analysis was performed using GeneMANIA plugin in Cytoscape with default setting [6]. The modEnrichr was used for ortholog conversion of identifiers from zebrafish (*Danio rerio*) to identifiers in human (*Homo sapiens*) [4].

**References**

1. Ansai S, Hosokawa H, Maegawa S, Kinoshita M. Chronic fluoxetine treatment induces anxiolytic responses and altered social behaviors in medaka, *Oryzias latipes*. Behav Brain Res. 2016;303:126-36.

2. Gupta T, Mullins MC. Dissection of organs from the adult zebrafish. J Vis Exp. 2010(37): e1717.

3. Chen EY, Tan CM, Kou Y, Duan Q, Wang Z, Meirelles GV, et al. Enrichr: interactive and collaborative HTML5 gene list enrichment analysis tool. BMC Bioinformatics. 2013;14:128.

4. Kuleshov MV, Diaz JEL, Flamholz ZN, Keenan AB, Lachmann A, Wojciechowicz ML, et al. modEnrichr: a suite of gene set enrichment analysis tools for model organisms. Nucleic Acids Res. 2019;47(W1):W183-90.

5. Kuleshov MV, Jones MR, Rouillard AD, Fernandez NF, Duan Q, Wang Z, et al. Enrichr: a comprehensive gene set enrichment analysis web server 2016 update. Nucleic Acids Res. 2016;44(W1):W90-7.

6. Warde-Farley D, Donaldson SL, Comes O, Zuberi K, Badrawi R, Chao P, et al. The GeneMANIA prediction server: biological network integration for gene prioritization and predicting gene function. Nucleic Acids Res. 2010;38(suppl_2):W214-20.
